# Commentary: Knowledge Acquisition during Exam Preparation Improves Memory and Modulates Memory Formation

**DOI:** 10.3389/fnbeh.2016.00245

**Published:** 2017-01-04

**Authors:** Emma L. James, Scott A. Cairney

**Affiliations:** Department of Psychology, University of YorkYork, UK

**Keywords:** memory, learning, schemas, hippocampus, development, sleep

New memories are rarely formed as individual representations, but are stored in the context of cognitive schemas: mental frameworks within which we can organize and interpret related information (Bartlett, 1932). At school, for example, we acquire basic knowledge about the locations of different countries. This geographical schema then assists us later in life when learning about new countries, their locations, and cultural similarities. As our global knowledge grows, the learning and integration of new geographical information becomes increasingly efficient, and we can make inferences regarding the world around us.

Schematic influences on learning can be understood in the context of dual systems models of memory (e.g., van Kesteren et al., 2012; McClelland, 2013). These models propose that new information is rapidly encoded by hippocampal networks, but becomes gradually integrated into the neocortex for long-term storage. However, where this new information is related to a schema, it benefits from existing neural connections and can thus be integrated more efficiently than unrelated information.

Several recent studies have examined the neural mechanisms underlying schematic learning by generating artificial schema in the laboratory (e.g., Hennies et al., 2016). While informative and well-controlled, this method is unable to address how we utilize real-world knowledge that is not bound to the context of an experiment. Understanding whether the neural mechanisms underpinning schema-based learning are similar in relation to natural variations in existing knowledge remains a key research question. Gaining insights into the automaticity with which we can capitalize upon our existing knowledge in different settings from which it is acquired may have important implications for education.

In a recent study, Brod et al. (2016) capitalized upon the advancing knowledge of medical students to gain more naturalistic insights into the neural basis of schema-relevant learning. While undergoing fMRI, participants learned a series of faces paired with either names or medical diagnoses (low or high relevance to medical knowledge, respectively). Immediately afterwards, outside of the scanner, they were re-presented with each face and asked to identify the associated name/diagnosis. This procedure enabled Brod et al. to identify patterns of neural activity at learning that predicted successful memory retrieval at test. Furthermore, this procedure was carried out with novel pairs both before and after 3 months of study for a medical exam, providing a measurable change in medical knowledge across the two time points. Brod et al. hypothesized that this increase in knowledge would reduce learning demands of the anterior hippocampus (AHc), a region that binds multiple neural inputs into integrated memory representations (Davachi, 2006).

Indeed, AHc activity decreased during successful schema-relevant (vs. schema-irrelevant) learning between the first and second time points. Two further analyses supported the role of schematic knowledge in driving this change. First, the decrease in AHc activity was positively correlated with overall gains in medical knowledge. Second, functional connectivity between the AHc and left middle temporal gyrus, a key semantic knowledge region (Binder et al., 2009), increased across time points, but only for schema-relevant learning. Collectively, these findings suggest that the AHc is less engaged in forming new memories when there is more schematic knowledge to support learning.

Brod et al.'s approach will be particularly valuable in developmental research, where understanding how poor levels of existing knowledge affect further learning is a crucial issue (Stanovich, 1986). Previous work has suggested that the AHc undergoes substantial development throughout the school years (Ghetti and Bunge, 2012). Anatomically, Gogtay et al. (2006) showed that AHc volume decreases between 4 and 25 years of age, and this structural change has been recently linked to children's increasing propensity for binding elements across multiple learning episodes (Schlichting et al., 2016). Functionally, Ghetti et al. (2010) observed age-related increases in AHc engagement during memory encoding. These concurrent decreases in anatomical volume and increases in functional activity may be best conceptualized as a fine-tuning of AHc learning mechanisms throughout development (Schlichting et al., 2016). Understanding how such changes affect the automaticity with which children access schematic knowledge will have important implications for memory theories and educational practice. Incorporating schema-consistent learning into teaching strategies could facilitate learning in the classroom, and drawing more explicit connections to existing knowledge may help to bring this benefit to children earlier in development.

Combining realistic, schema-based learning paradigms with sleep research will also provide new insights into the neurobiological mechanisms of memory. Sleep supports memory consolidation (Rasch and Born, 2013), and slow-wave sleep (SWS) in particular has been linked to the integration of memories within schematic knowledge (Lewis and Durrant, 2011). Research on this topic has been primarily focused on memory retrieval (Hennies et al., 2016). Given the evidence that prior schematic knowledge facilitates learning (Brod et al., 2016), it will be important to understand how AHc engagement at encoding impacts on the subsequent role of sleep in memory integration. Interestingly, children exhibit a higher proportion of SWS than adults (Ohayon et al., 2004), and this has been linked to a stronger capacity for overnight memory consolidation (Wilhelm et al., 2013). In the context of schema-based learning, enhanced SWS in childhood might support the integration of new information into existing knowledge where AHc encoding mechanisms are not yet fully developed.

The periods of intensive study typically required for experimental manipulations of schematic knowledge raise a number of potential ethical concerns in developmental settings: they are time-demanding, resource-intensive, and involve learning material irrelevant to the curriculum. The paradigm offered by Brod et al. provides developmental neuroscientists with a practical solution for these issues, whereby school topics can be incorporated into studies of schema-based learning. Although this approach does not benefit from the experimental control offered by laboratory-based studies, it would enable assessments of large groups of children, reducing demands on additional learning time while gaining fundamental insights into real-world memory processes.

In summary, Brod et al. (2016) demonstrated that schematic knowledge reduces AHc demands during memory formation in the context of real-world learning. These findings raise important questions regarding learning mechanisms where the AHc is still undergoing development, and provide practical directions for studying these mechanisms in educational settings.

## Author contributions

EJ and SC contributed equally to conceptualizing and writing the manuscript.

### Conflict of interest statement

The authors declare that the research was conducted in the absence of any commercial or financial relationships that could be construed as a potential conflict of interest.
